# Inefficient Eye Movements: Gamification Improves Task Execution, But Not Fixation Strategy

**DOI:** 10.3390/vision3030048

**Published:** 2019-09-18

**Authors:** Warren R. G. James, Josephine Reuther, Ellen Angus, Alasdair D. F. Clarke, Amelia R. Hunt

**Affiliations:** 1 School of Psychology, University of Aberdeen, Aberdeen AB24 3FX, UK; w.james@abdn.ac.uk (W.R.G.J.); j.reuther@abdn.ac.uk (J.R.); e.v.angus.14@aberdeen.ac.uk (E.A.); 2 Department of Psychology, University of Essex, Colchester CO4 3SQ, UK; a.clarke@essex.ac.uk

**Keywords:** visual search, eye movements, attention, strategy, decision

## Abstract

Decisions about where to fixate are highly variable and often inefficient. In the current study, we investigated whether such decisions would improve with increased motivation. Participants had to detect a discrimination target, which would appear in one of two boxes, but only after they chose a location to fixate. The distance between boxes determines which location to fixate to maximise the probability of being able to see the target: participants should fixate between the two boxes when they are close together, and on one of the two boxes when they are far apart. We “gamified” this task, giving participants easy-to-track rewards that were contingent on discrimination accuracy. Their decisions and performance were compared to previous results that were gathered in the absence of this additional motivation. We used a Bayesian beta regression model to estimate the size of the effect and associated variance. The results demonstrate that discrimination accuracy does indeed improve in the presence of performance-related rewards. However, there was no difference in eye movement strategy between the two groups, suggesting this improvement in accuracy was not due to the participants making more optimal eye movement decisions. Instead, the motivation encouraged participants to expend more effort on other aspects of the task, such as paying more attention to the boxes and making fewer response errors.

## 1. Introduction

We use eye movements to solve many problems, such as avoiding obstacles, or to find an item of importance. The ability to direct our vision is invaluable. In some situations, humans appear able to use eye movements to direct the visual system in an optimal manner [1]. In other situations, people do not make eye movements that are consistent with an optimal strategy [2,3]. Najemnik and Geisler [1] derived an “ideal observer” model of eye movements in visual search, which minimises the number of fixations needed to find a target by deploying fixations that reduce overall uncertainty of information in the search space. To do this, the model accounts for fixation history, as well as differences in acuity across the retina, to select fixations that provide the most new information. They found that humans matched the ideal model in terms of the number of fixations needed to find the target, suggesting that we make decisions about where to fixate based on maximising information gain. However, calculating expected information gain for all possible fixations is a resource-intensive process, considering we tend to select new fixations 2–3 times each second during search. With this in mind, Clarke et al. [4] suggested a simpler alternative model, i.e., human performance in a visual search task can be characterised as a stochastic process. The stochastic model randomly selects each eye movement during search from a population of saccade vectors executed from that region of the search array, until the target is “found” (that is, until the model’s fixation lands close enough to the target that it would be detected, given empirical estimates of target visibility over eccentricity). Though the population from which the saccades were selected may have been shaped by factors such as the target visibility and search history, the model itself did not use this information in selecting each new fixation location. Similar to Najemnik and Geisler [1], Clarke and Hunt [2] also found that their model matches human observers in terms of the number of fixations needed to find the target, despite the selection process being random instead of driven by expected information gain.

This leaves us with a puzzle: two models with very different underlying mechanisms that both describe human search equivalently well. On the one hand, humans may make each fixation based upon current scene statistics and fixation history, and, on the other hand, their fixation selection may be based on a more generic set of perceptual and motor biases. The assumption that differentiates these two models is whether fixations are selected based on a principle of expected information gain.

Experiments by Nowakowska et al. [5] directly test this assumption and suggest both models may be correct. They presented participants with search arrays that had two halves: on one half, the target would pop out from the distractors and could be easily seen using peripheral vision, and on the other side, the distractors were more variable and the target would be harder to spot. An optimal searcher would direct all fixations to the more difficult side, because no new information would be gained by fixating on the easy side. The results show large individual differences. A few participants conformed to an optimal strategy, but a wide range of other strategies were observed. These strategy differences were closely correlated with the efficiency of search performance, and Clarke et al. [6] showed they were also stable over time (see also [7]). These studies suggest that no single model can describe fixation selection during search: variability driven by contexts and individuals will need to be accounted for.

Another paradigm that addresses the extent to which eye movements are driven by expected information gain was devised by Morvan and Maloney [3]. In their task, participants had to make a single eye movement in order to discriminate a small target dot that could appear in one of two locations. In the beginning of each trial, participants were presented with three boxes: one in the centre, with two boxes either side. Participants were instructed to fixate one of these boxes and then the target would appear in either of the two side boxes. Throughout the experiment, these two side boxes would appear at different eccentricities, sometimes being placed very close to the central box, and at other times a large distance away. When the boxes were closely spaced, the optimal strategy would be to fixate the central box, using peripheral vision to detect the target when it appears to the left or right of fixation. However, as the distance between the boxes gets larger, performance from a central fixation position begins to decrease towards chance (50%). Therefore, for a big enough distance, the optimal strategy is to fixate either of the two side boxes, as this would achieve an expected accuracy of around 75% (100% for targets appearing in the fixated box, and 50% for targets appearing in the other box). If participants were to select fixations according to expected information gain, given the limitations of their own visual acuity, they would fixate the centre box when expected accuracy given the distance between the boxes is greater than 75%, and one of the side boxes when expected accuracy from the centre is less than 75%. The results of Morvan and Maloney’s experiment clearly demonstrate that people failed to select fixations according to this optimal strategy. This failure went well beyond not quite switching at the optimal point, or not consistently carrying out the optimal strategy. In fact, none of the participants in their study modified their choice of which box to fixate according to the distance between the boxes.

This failure to fixate in a way that optimises information gain during search does not appear to be a limitation that is specific to eye movements and visual search. Clarke and Hunt [2] demonstrated the same striking decision failure across a range of different tasks (like choosing where to stand to throw objects at targets, or choosing whether to try and memorise one or two digit strings for later report). They characterised the decision failure as an inability to use an expectation of task difficulty to guide decisions about whether to attempt to accomplish multiple goals or to focus resources on a single task.

Sub-optimal behaviour is prevalent in many other tasks [8]. Potential explanations for the observed patterns were discussed by Kahneman and Tversky [9], as well as Gigerenzer and Gaissmaier [10]. One important consideration in the decision dilemma posed in the current study is that participants might not have an accurate representation of their own ability, and, hence, are unable to adopt the optimal strategy, which requires them to use that information to decide whether to pursue one goal or two. This would be an example of Bounded Rationality [11] where human behaviour is argued to be optimal given some constraints. The constraints in this case would come from imprecise information about one’s own capabilities, or a lack of confidence in the accuracy of that information. This explanation seems unlikely, because participants in these experiments first complete a session in which their ability to perform the task was recorded (e.g., throwing, memorising or detecting), without the need to make a decision. The purpose of this session is to calculate their switch point in the second session, and to calibrate the second session’s difficulty level to match the capability of the participant, but a secondary benefit of this session is that the participant gains experience doing the task and observing their own chances of success at different levels of task difficulty. To check if participants successfully learned this information, James et al. [12] asked participants to provide estimates of their chances of successfully hitting a target at a range of different distances. They found that participants’ estimates were very close to their actual accuracy in performing the task. This suggests that participants do have the information necessary to perform optimally, but do not make use of this when making decisions.

Other explanations for suboptimal decisions in tasks like this could be based on other classic cognitive biases, such as risk/loss aversion [9] or achievement motivation [13]. However, there is a great deal of variation in the decision behaviour of individual participants, with some consistently trying to accomplish both goals, and others consistently focusing on one, and most showing a blend of both strategies (but rarely in a way that varies systematically with task difficulty). As such, one single bias or heuristic might be able to explain the behaviour of some individuals on some trials, but does not provide a satisfying explanation for sub-optimal performance in the experiment overall.

The potential explanation for sub-optimal performance that is addressed in the current experiment is a lack of motivation. The idiosyncratic and variable decisions the participants tend to make in this paradigm may be due to participants prioritising something other than accuracy in the task when making their decisions. Some other studies that added a financial incentive have shown that participants begin to make use of more optimal strategies [14,15]. A clear motivation for success in the form of a financial gain can lead participants to move from employing strategies that attempted to be successful for all outcomes to focus more on those that were more likely. It is important to note that the participants in the experiments of Morvan and Maloney [3] (*N* = 4 in Experiment 1, *N* = 2 in Experiment 2) were financially rewarded for correct responses, up to a total of $20. However, the monetary gain was spread out over thousands of trials, which may have diluted its effects. In Clarke and Hunt [2], there were more participants and the experiments were much shorter, but participants were offered no monetary reward for being successful, relying instead on the value placed on success by each participant (which may in some cases have been non-existent). Therefore, it is possible that at least some of the participants were simply not motivated to succeed.

Even with an added financial incentive, the goals of a participant may still be misaligned with the desires of the experimenter. For example, even though in Morvan and Maloney [3] participants were rewarded for success, they may have prioritised completing the task as quickly as possible rather than gaining the largest sum of money at the end of the experiment. This could be the result of participants wanting to maximise the rate of gain over time as opposed to solely focussing on the total gain. Miranda and Palmer [16] suggested that game-like experiments can encourage participants to prioritise success rather than speed of completion. They posited that enjoyable experiments intrinsically motivate participants to focus on the task as opposed to simply performing the task to gain something external (such as money or course credit). In effect, this could align the goals of the participant and experimenter as participants would find the task more enjoyable and be less likely to rush through it or be negligent.

In the present study, we gamified the decision problem with the aim to motivate participants to adopt a more success-oriented attitude. We hypothesise that participants taking part in a more engaging version of the task would be more likely to use a more optimal strategy, resulting in a greater rate of success.

## 2. Materials and Methods

### 2.1. Participants

Participants (*N* = 18, 13 female) were recruited via word of mouth at the University of Aberdeen. The age range was 20–23 (mean of 20). The *Control* group data (*N* = 12) and *Optimal* group data (*N* = 12) were drawn from another study using the same paradigm but without a game context (Experiment 3 in [17]).

### 2.2. Procedure

The experiment took part in one session with two parts. The aim of the first part was to measure each participant’s visual acuity in order to tailor the second part to each individual. The first part lasted approximately 30 min. In the second part, participants carried out the decision task, which lasted between 40 and 50 min.

Both parts made use of an Eyelink 1000 (version 4.594, SR Research ltd, Mississauga, ON, Canada), which recorded eye position at 1000 Hz. Participants were seated ≈ 53 cm from the screen, which was maintained throughout the experiment by the use of an adjustable chin rest. A Sony Trimaster EL OLED was used to display the stimuli. The resolution was 1920 × 1080 p (54 cm wide, 31 cm tall) with a refresh rate of 60 Hz. The experiment was programmed for and run in Matlab 7.9.0 (R2009b) with Psychtoolbox [18] and EyelinkToolbox functions [19]. Prior to starting each part, a 5-point calibration was carried out. The calibration sequence was repeated at the start of each block. Additionally, calibration was triggered by a failure to fixate cumulatively for 10 times since the last calibration, or if there were 5 fixation errors in a row.

### 2.3. Visual Acuity

**Figure 1:**
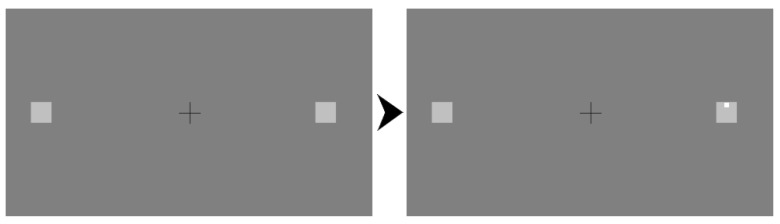
The setup for the visual acuity task. Each trial began with the elements in the (**left**). After the central fixation crosshair was fixated for 700 ms, the discrimination target appeared (shown in the right box in the (**right**)).

In Part 1, participants were presented with a fixation cross with two boxes placed either side (Figure 1). They were told that their task was to identify whether a small dot (≈ 0.3°) had appeared in the top or the bottom half of one of the boxes (each ≈ 1°), irrespective of the box it had appeared in, by pressing the “up” or “down” arrow, respectively, on the keyboard. The dot had an equal chance of appearing in either box and in either position. To commence each trial, participants had to press the spacebar whilst fixating the central cross. After maintaining fixation for 700 ms, the target dot would appear in one of the two boxes, at random, and was displayed for 500 ms. If participants broke fixation during any stage of this process, the screen would turn red for 1 second to indicate that they had broken fixation.

The boxes were presented at varying distances from the central fixation cross (2.7°, 3.9°, 5.2°, 6.8°, 8.4°, 10.1°, 11.4°, and 12.5°). There were four blocks of 96 trials each. Each distance was presented 12 times in a pseudo-random order where all 12 trials per distance were presented in succession. Data from Part 1 were then used to calculate the distance at which participants were 75% accurate in detecting the target. This value was subsequently used in the second part to tailor the experiment to each individual.

### 2.4. Decision Task

**Figure 2:**
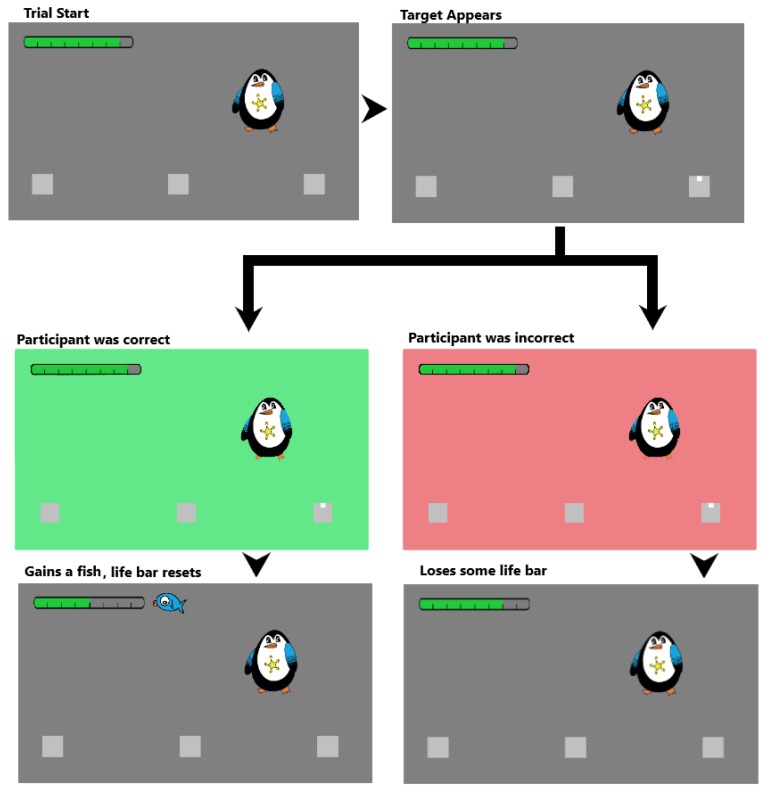
An example trial sequence where the life bar starts one “tick” away from the maximum. A correct response gains the participants a fish reward and the bar returns to the mid-point (**left**). An incorrect response leads to a loss of one “tick” from the life bar (**right**).

For the second part, participants were introduced to Pugadoo the Penguin. Pugadoo was approximately 5.7° tall and 4.9° wide, and had a star on his belly, which served as the fixation mark. Participants were told that their task was to collect as many fish as possible for Pugadoo. To do so, they needed to choose one of three boxes, fixate it and correctly report whether the target dot was presented in the upper or lower half of one of the two side boxes (Figure 2). Participants were told they could fixate any one of the three boxes, but that the target would only ever appear in one of the two side boxes (selected at random). On each trial, Pugadoo was placed in the top half of the screen so that the star on his belly would end up either midway between the centre and the left, or the centre and the right box, respectively (with an equal chance). This placement matched the placement of the fixation cross used in the comparison set of data [17]. The three boxes were placed along the horizontal meridian, one box in the centre of the screen, and two boxes equidistant on either side of the central box. Boxes were presented with one of nine distances, of which seven were based on each participant’s individual switch point that was calculated based on each participant’s performance in the first part. These distances were defined as the switch point itself, and then the switch point ±3°, ±2°, and ±1°. A further two distances were constant across all participants (8° and 18°). In the left corner of the screen, participants were presented with a bar indicating the number of correct responses needed to earn the next fish. The bar was presented in the top left corner of the screen throughout the duration of the task, and was approximately 12° by 0.75° in size. The bar had eight “ticks” and started half way filled (four ticks) at the beginning of each block. One tick was added, or deducted, for each correct or incorrect response, respectively. If the bar was filled to eight ticks, one fish would be added to the right hand side of the life bar. If the bar decreased to have 0 ticks, then the number of fish would decrease by one. After a change in fish count, on the subsequent trial, the bar would reset to being half filled. Fish were presented next to the bar and remained visible for the duration of the block.

At the beginning of each trial, participants would fixate the star on Pugadoo’s belly (Figure 2) and press the spacebar. After a stable fixation of 700 ms, three boxes were displayed on screen. Participants then executed a saccade to the box of their choice (left, centre or right). After 50 ms of a stable fixation in the selected box, a target dot would appear in one of the side boxes for 500 ms. Participants indicated its position within the box (top or bottom) by pressing the up or down arrow keys. If the response was correct, the background turned green and the bar would fill by one tick. If the response was incorrect, the background would turn red and the bar would be emptied by one tick. Filling the bar completely was accompanied by a sound cue of Homer Simpson celebrating in his traditional manner of “Woohoo!” to signify that they had gained a fish. When fully depleting the bar, a fish would be taken away from the total at which point a separate sound cue was presented (Homer Simpson saying “Doh!”), signifying the loss of a fish. If the participant had gained or lost a fish, the life bar would be reset to being half filled. The constant presentation of the bar and the fish, together with the trial-by-trial feedback allowed participants to monitor their performance while completing the task.

### 2.5. Comparison Data

Additional data were retrieved from https://osf.io/t6c5q/ in order to compare the current study’s data to that of non-motivated participants (*Control* group, *n* = 12) and of participants that were guided as to where they should fixate (*Optimal* group, *n* = 12). The setup for the comparison data was the same as in the current study (for specificities see [2]). However, instead of Pugadoo, participants were presented with a fixation cross (as in Figure 1). The *Control* group carried out the task as per Clarke and Hunt [2], which is identical to the current study. To enforce the use of the optimal strategy, the *Optimal* group was instructed which box to fixate, on a trial by trial basis. To indicate the optimal box, the respective box would blink. This box was determined based on the individual participants’ performance in the first part. That is, the central box would blink if their expected accuracy for the current box distance was above 75%. Otherwise, one of the side boxes would blink (side chosen at random). Trials where participants failed to fixate the optimal box were aborted.

These acted as comparison groups for the current study; if motivation does encourage people to make more optimal decisions, then the participants in the current study should differ from the *Control* group and be more similar to the *Optimal* group. In the original study, participants completed eight blocks of decisions. However, the *Optimal* group were only guided for the first four blocks. As such, only the first four blocks for each participant were used in the analysis section here. Additionally, the current study only used four blocks and as such taking the first four blocks ensures that any learning effects are equalised due to the same level of experience with the task. Part 1 was the same for all three groups.

### 2.6. Analysis

All analyses were carried out in R version 3.4.3 [20] using rStan version 2.16.2 [21]. (Scripts for analysis and all data can be found under https://osf.io/kh2sy/, as well as scripts for producing plots and processing the data, which made use of Tidyverse version 1.1.1 [22]). To model these data, we used a Bayesian beta regression model [23] with logit link in order to compare the different rates of success between the three groups. The reason for using a Bayesian approach is that we are more interested in looking at the effect size, and associated variance and uncertainty, than examining whether the difference is significantly different from zero. We opted to use Beta regression rather than logistic regression, as this allowed us to directly compare the observed rates of success with each participant’s expected rate of success (which is explained below).

**Figure 3:**
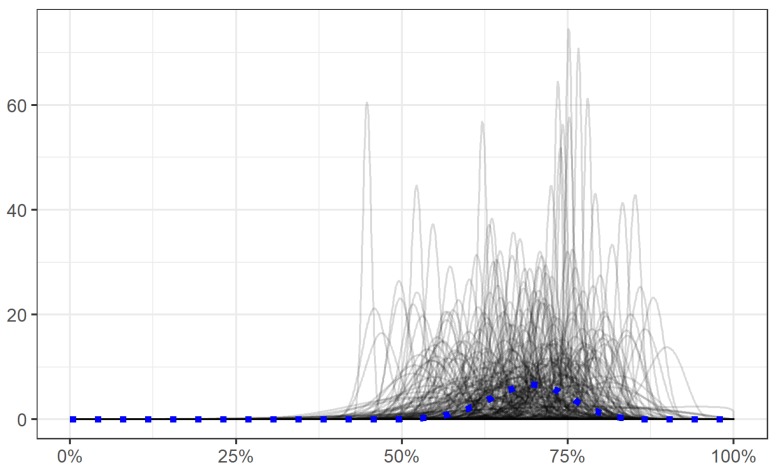
This plot shows 250 samples from the prior in black. The dotted blue line shows the prior using the values obtained from Part 1 data.

A prior was calculated for the *Control* group based on the performance of participants in Part 1. To estimate these, a Beta distribution was fit to the average accuracy data for all participants in Part 1. Measurements for the mean and precision were calculated from the fit shape parameters, and the appropriate transformations applied in order to set the priors for the linear component of the model. The prior for the coefficient for the mean (0.8; SD = 0.4) was normally distributed, as was the coefficient for precision (5.6; SD = 1.4). The mean values for the priors were directly calculated from the Part 1 data, whereas the standard deviations were set to allow a plausible range of beta distributions (see Figure 3). Several samples from these priors can be seen in Figure 3. We used weakly informative Priors (a normal distribution with mean = 0, SD = 1) for the effect of being in the *Motivated* and *Optimal* group.

## 3. Results

### 3.1. Fixation Proportions

On each trial, participants were given three possible locations to fixate, and the target did not appear until a fixation was detected in one of these locations. Fixations were coded as being either a central fixation (i.e., they fixated the centre box), or as a side fixation (i.e., they fixated one of the side boxes). A proportion was then calculated for how often the participant fixated one of the side boxes as a function of box distance.

**Figure 4:**
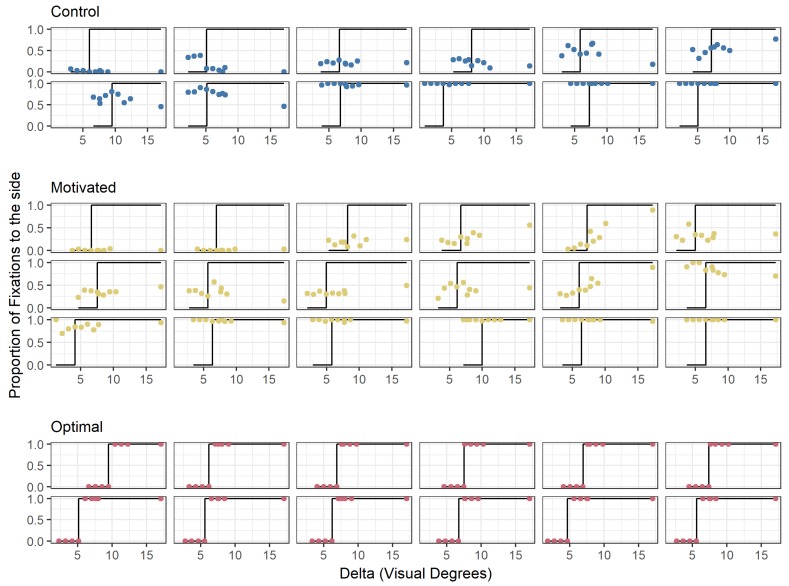
Plot of proportion of fixations to the side box (*y*-axis) with increasing distance between the centre and side boxes (Delta, *x*-axis). Each separate plot shows the behaviour for an individual participant: **bottom** (in red) the behaviour of participants who were guided to employ an optimal fixation strategy; **middle** (in yellow) the *Motivated* group; and **top** (in blue) the *Control* group. The black line shows where participants would have fixated, had they made use of the optimal strategy.

Figure 4 shows the proportion of fixations to one of the side boxes for each Delta (distance of the side boxes from the central box in degrees of visual angle) that was tested for each participant. This is split to show the different conditions under which the participants completed the task, with one panel for the data of the *Control* group, one panel for the data of the *Motivated* participants, and one panel for the participants who were guided to make optimal decisions. From an initial inspection of Figure 4, participants in the *Motivated* group do not appear to differ from the *Control* group. Both, the *Control* group and the *Motivated* group differ dramatically from the *Optimal* group, who were instructed where to fixate on each trial. In Figure 4, the plots have been arranged by the proportion of fixations each participant made to the central box. This helps to demonstrate that there is a wide range of behaviours that can be, and are, observed in these data. There does not appear to be any systematic difference between the *Motivated* and *Control* group. Instead, the *Motivated* group are as variable as their counterparts in the *Control* group.

### 3.2. Rate of Success

Initial analysis for rate of success examined the overall accuracy for each participant. To do this, an average was calculated for each participant, which was then modelled as described above. For each model, the average value from the posterior are reported as well as the 95% Highest Posterior Density Interval (HPDI) for this value. The HPDI is the narrowest region of the posterior distribution that contains, in this case, 95% of the estimates for the mean.

Rate of success was modelled using group as a predictor variable. As the primary interest of this paper is the overall rate of success, models including Delta (Visual Angle) are not included in the main text, but are available in the Supplementary Materials (https://osf.io/kh2sy/).

**Figure 5:**
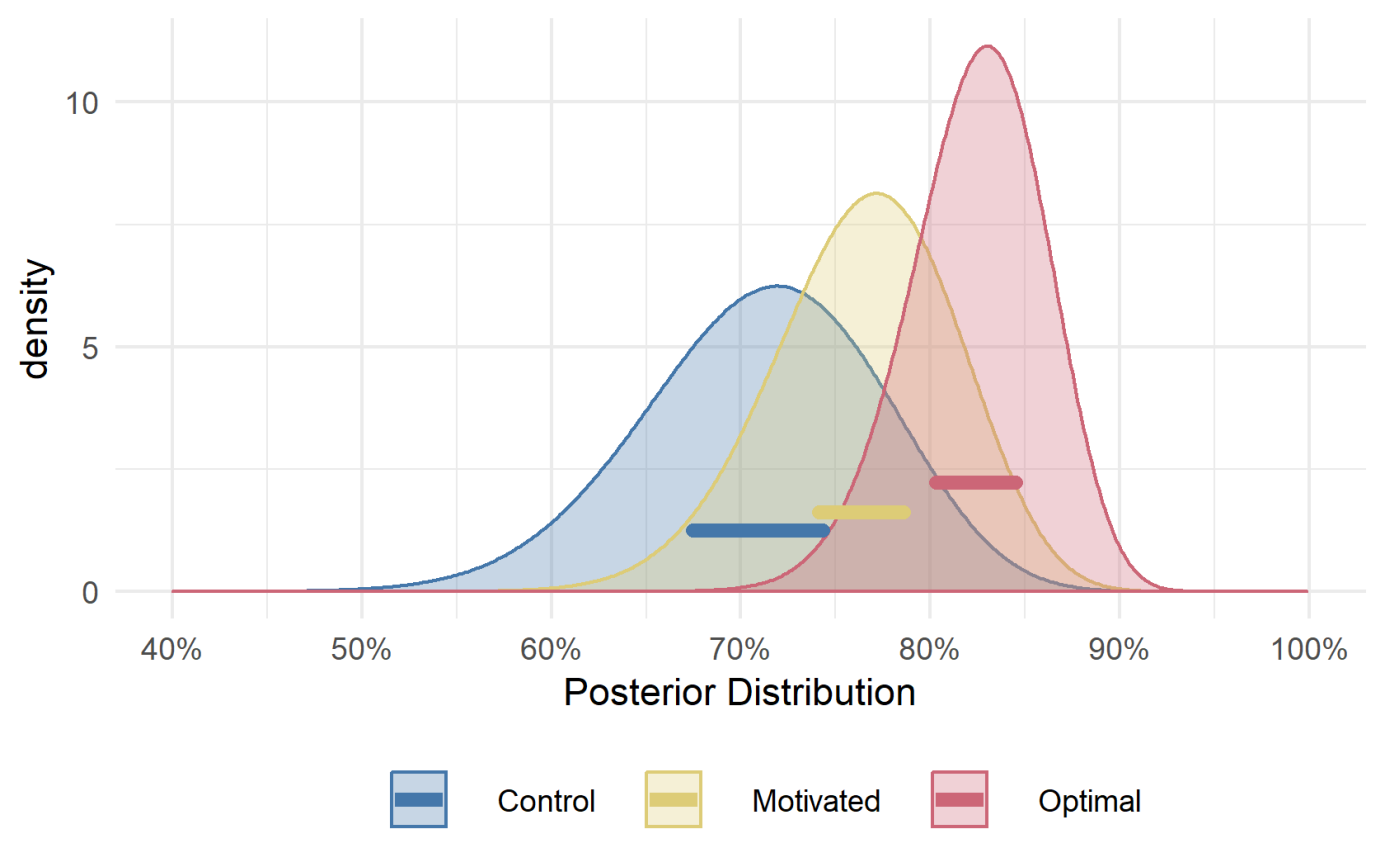
Posterior distributions of success rate in target detection. The lines highlight the 95% HPDI for the mean of each group’s predicted rate of success.

Figure 5 shows the posterior distributions for success rate in target detection. The *Motivated* group were on average 5.4% (95% HPDI of |1.4%, 9.4%|) more successful than the *Control* group. Neither group managed to reach the same rate of success as the *Optimal* group who were on average 11.3% (95% HPDI of |7.3%, 15.5%|) more successful than the *Control* group, and 5.9% (95% HPDI of |2.7%, 9%|) more accurate than the *Motivated* group. This suggests that our manipulation was effective: while the *Motivated* group still behave sub-optimally, they do manage to outperform the *Control* group. The results also demonstrate that the optimal fixation decision strategy does substantially improve success (i.e., detection accuracy), and that the *Motivated* group, while better than the *Control* group, does not achieve the detection success rate of the *Optimal* group.

### 3.3. Expected Rate of Success Given Fixation Strategies

The higher rate of success in the discrimination task for the *Motivated* group relative to the *Control* group may initially be surprising, given the lack of any clear differences in the fixation strategies between the *Motivated* and *Control* group that can be observed in Figure 4. Given that the distances in the decision task were calibrated to each individual’s performance in Part 1, the same overall accuracy can be expected across groups if their fixation strategies are similar. The likely explanation is that the introduction of Pugadoo in the second part of the experiment caused the *Motivated* group to try harder to see the dot, and to make more careful responses in the discrimination task, relative to the *Control* group, who performed the task without any additional incentive to respond correctly.

To investigate what proportion of the differences between groups could be explained by a more optimal fixation strategy, rather than general improvements in the discrimination task, each participant’s expected success rate was calculated given the fixations choices they had made on each trial. This calculation of expected success was based on each participant’s Part 1 performance, before the penguin character was introduced to the *Motivated* group. Using the participants’ performance in Part 1, it was possible to estimate how likely each participant was to detect the target at all possible distances that were presented in Part 2 by fitting a psychometric function to the data (This was carried out using the Psyphy version 0.1–9 [24], which allowed for the chance level to specified at 50%). To get a measure of expected accuracy, we calculated the participants accuracy for the box on the left ($B_{l}$) and the box on the right ($B_{r}$) given their distance from fixation. Both of these values were then multiplied by the chance (*c*) that the target would appear in that box, and then summing these values. In this setup, each box was equally likely to contain the target, and as such were both multiplied by *c*, which equalled 50%. That is, for far apart boxes, a participant who fixated the left box would have a 100% chance of success for $B_{l}$, and a 50% chance for $B_{r}$, as at this distance the target is imperceptible and their chance is equal to that of guessing. In this case, the expected chance of success would be 75%, which is given by calculating ($B_{l}$ × *c*) + ($B_{r}$ × *c*). This estimate of accuracy, given the actual fixation choice, was calculated for each participant and trial, and then averaged over all trials per participant to get their individual rate of success. This “estimated success rate” removes the effect of improvements in discrimination performance from Part 1 to Part 2, and also removes the effects of any runs of good or bad luck for any specific participants (e.g., choosing the target or non-target side on more than half the trials, or seeing the target but pressing the wrong key). As such, this measure provides an estimate of the effect on success rate of fixation decisions alone; any improvements to performance in the *Motivated* group compared to *Control* group in this measure will reflect a more effective fixation strategy.

**Figure 6:**
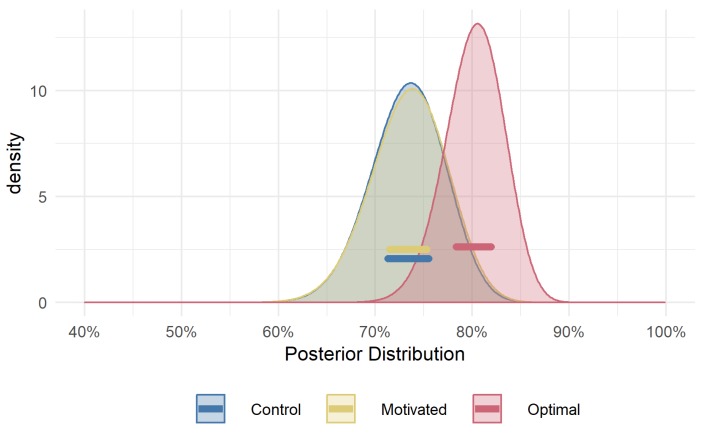
Posterior distributions of estimated success rate in target detection given fixation decisions. This estimate of success is based on each participant’s visual acuity as measured in Part 1, thus isolates the effect of fixation decisions on detection performance. The lines highlight the 95% HPDI for the mean of each group’s expected rate of success.

**Figure 7:**
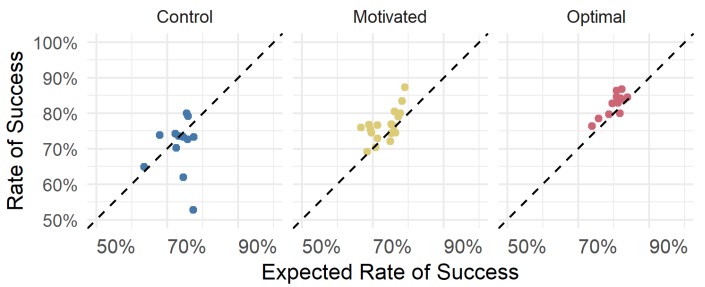
These scatter plots show a point for each participant’s actual rate of success and how it relates to their expected rate of success. The dashed line is a line with slope 1 going through the origin which would represent a 1:1 relationship (i.e., they performed as well as would be expected).

It is clear from the results in Figure 6, which shows the posterior distributions for expected success rate, that the *Control* and *Motivated* group are now almost perfectly overlapping. In other words, the difference that was observed when modelling participant’s raw success rate (Figure 5) entirely disappeared: the difference between the average expected rate of success between the *Motivated* and *Control* group was ≈0% (95% HPDI of |−0.25%, 0.3%|) suggesting that the *Motivated* and *Control* group were very similar, if not identical, in terms of their expected success rate. These results suggest that the previously observed greater rate of success exhibited by the *Motivated* group was not due to them making better, more strategic decisions about where to fixate. Instead, this greater rate of success is due to other factors, such as trying harder to see the target and trying not make any button-pressing errors (or perhaps they were simply more lucky than their non-motivated counterparts). As can be seen in Figure 7, the *Motivated* and the *Optimal* group appear to perform the task in such a way as to reach a similar level of success as would be expected given their choices; however, the *Control* group appears to have some participants whose performance falls short of what would be expected.

## 4. Discussion

The results from this experiment are consistent with previous findings in that participants are highly variable and suboptimal in their fixation decisions in this task [2,3]. Despite the presence of a strong motivation to be optimal, which demonstrably increased participants’ efforts to do well in the task, none of the participants adopted the optimal strategy. The novel contribution from the current experiment is that the decisions about where to fixate (Figure 4) show no discernible difference in strategy between the *Motivated* and the *Control* group. This would suggest that having a clear motivation for success does not cause people to use more optimal strategies. This result rules out a failure to adequately motivate participants as a likely explanation for suboptimal decisions.

The *Motivated* group did have higher overall accuracy in the task than the *Control* group, demonstrating that the motivation manipulation was effective. The boost in accuracy in the absence of an improved strategy suggests participants were expending more effort to detect the target and respond correctly. This result is consistent with several studies demonstrating that visual sensitivity, attention, and effort are all affected by the presence of rewards [16,25,26]. Participants in the *Motivated* group may have made fewer erroneous button presses (that is, pressed the key they intended to) than the *Control* group as a result of more sustained attention/lower lapse rates [27]. They also may have made more of an effort to detect the target during the decision phase, in which the “game” was introduced, and therefore were better able to detect the target than would be expected given their initially measured visual acuity [16].

This finding rules out a general lack of motivation as an explanation for why participants fail to discern or follow an easily implemented decision rule that would increase their accuracy. This can be added to a growing list of other simple explanations. As noted in the Introduction, James et al. [12] ruled out a lack of self-awareness of ability as a viable explanation. Another possible explanation is that the optimal strategy may in fact be more difficult to implement than it initially seems. Hunt et al. [17] ruled this out as well by asking non-naive participants to perform both the Throwing and Detection task from Clarke and Hunt [2]. The results from these participants were very close to optimal. This demonstrates that it is possible to implement the strategy when it is known. However, calculating optimal fixations in the context of visual search can be quite effortful (as demonstrated by the ideal search model of Najemnik and Geisler [1]), and does not always result in large gains in terms of utility or efficiency relative to making “random” fixations [4]. This may go some way towards explaining why participants do not employ the optimal strategy in the simpler context of the current experiment. That is, participants may not know *a priori* that the strategy in this particular case is simple and will benefit performance, but they may know that the gains that can usually be achieved by figuring out an optimal strategy would not outweigh the cost of figuring out the strategy in the first place. This could be considered an example of satisficing, in that participants reached a point at which their strategy met their own moderate standards [11]. Here, the participants of the *Motivated* group might have set themselves a standard for performance that they were able to meet without changing their fixation strategy: their goal was simply to get as many fish as they could. In deed, all of them completed the experiment with a net positive number of fish, which may have been sufficient for them to feel that they did their best. The presence of a motivating element presumably raised their standard above that of the *Control* group, and therewith their performance. However, had they explicitly recognised the additional gains they could have made with better fixation strategies, they may have re-examined fixation strategies and made more efficient decisions.

A striking contrast exists between the results from this task, in which participants are highly variable and sub-optimal, and examples from visually-guided reaching and eye movements for tasks in which participants appear able to execute near-optimal movement plans (e.g., [28,29]). In these studies, participants are able to take into account their own variability in movement production to target locations that balance risk and reward to optimise expected gain. The key difference between those experiments and the one presented here (and in [2,3,12,17]) is the directness of the relationship between the strategy and the rewarded outcome. In the current task, participants need to select a place to fixate that optimises their accuracy in the detection task, but it is performance on the detection task that is ultimately rewarded. This highlights an interesting distinction between optimal decisions about how to prepare for an upcoming task, relative to optimal decisions about how to execute the task when the time comes. Rather than the extent of motivation, it might be the directness between motivation and (optimal) strategy that needs to be higher for the latter to be adopted. In the current study, the execution of the detection aspect of the task responded to the motivation manipulation and showed clear improvements, but the decision about where to look in preparation for the task was unchanged. A similar dissociation is reported in [30], where participants performed rapid reaches towards pairs of lights, one of which was only revealed to be the target after the movement had begun. In these experiments, participants were able to weigh up possible movement plans and select those which minimised mechanical effort. However, they completely failed to flexibly adapt their reaching strategies to maximise expected success, similar to the decision failure reported in the current experiment.

Reinforcement learning is likely to play an important role in shaping optimal behaviour in the execution component of decision making scenarios. An interesting question for future research is to address why this reinforcement does not improve the strategic preparation aspects of these decisions.

## 5. Conclusions

The results of this experiment demonstrate that having a clear motivation to succeed does not facilitate the use of more optimal strategies when performing a task. The pattern of results obtained from the *Motivated* group are in line with those obtained in previous research. The improvement in their detection performance despite the failure to adopt better strategies demonstrate they are trying harder to successfully complete the task. In other words, motivation caused participants to “work harder, not smarter”.
